# Comparison of the Perioperative and Postoperative Effects of Levobupivacaine and of Levobupivacaine + Adrenaline in Pediatric Tonsillectomy: A Double-Blind Randomized Study

**DOI:** 10.1155/2017/8431823

**Published:** 2017-08-24

**Authors:** Faruk Cicekci, Cigdem Sizer, Sait Selcuk Atici, Sule Arican, Adnan Karaibrahimoglu, Inci Kara

**Affiliations:** ^1^Department of Anesthesiology, Selcuk University, Medical Faculty, Konya, Turkey; ^2^Department of Otolaryngology, Konya Numune Hospital, Konya, Turkey; ^3^Department of Statistic, Necmettin Erbakan University, Medical Faculty, Konya, Turkey

## Abstract

**Objectives:**

We aimed to assess the effects of levobupivacaine and of levobupivacaine + adrenaline administered during pediatric tonsillectomy on the postoperative period.

**Methods:**

A total of 90 patients between the ages of five and twelve were divided randomly into two groups before tonsillectomy: levobupivacaine only (0.5%) 0.4 mg·kg^−1^ or levobupivacaine (0.5%) 0.4 mg·kg^−1^ + adrenaline (1 : 200.000) administered by means of peritonsillar infiltration. Primary outcomes were postoperative pain scores recorded at various intervals until 24 hours postoperatively. Secondary outcomes included postoperative nausea and vomiting (PONV), time to first oral intake, time to the first administration of analgesics and total consumption of analgesics, and the amount of bleeding for all children.

**Results:**

In both groups, patients had the same postoperative pain scores and PONV rates, and equal amounts of analgesics were consumed up to 24 hours postoperatively. The two groups also had the same time until first oral intake, recovery time and time to the first analgesic request, and amount of bleeding.

**Conclusions:**

Perioperative levobupivacaine infiltration on its own is a valid alternative to the combination of levobupivacaine + adrenaline for perioperative and postoperative effectiveness in pediatric tonsillectomy. This trial is registered with Australian New Zealand Clinical Trial Registry ACTRN: ACTRN12617001167358.

## 1. Introduction

Tonsillectomy is among the most frequently performed surgical procedures for commonly seen occlusive and recurrent infections in otolaryngology [1]. Patients frequently complain of pain on swallowing after this operation [2, 3]. Furthermore, postoperative sore throat may cause adverse effects that decrease oral intake and lead to dehydration [4]. Sore throat also may cause certain late postoperative complications, such as epithelial loss in the surgical site and necrosis in soft tissue, bleeding in dehydrated patients, severe pain, and delayed recovery. Various tonsillectomy studies have shown the advantage of local anaesthetic (LA) injection, which often is performed before procedures to prevent pain stimulus during the operation [5]. The reasons for using LA agents perioperatively are both to block peripheral nociceptive excitation after tissue damage and to prevent the sensitization of the central nervous system [6]. LAs generally can be used as a preparation together with adrenaline, which has a very strong vasoconstrictor effect: (a) the addition of the LA to the blood is prevented, the LA remains in the surgical site, and the LA effect is maintained better [7]; (b) the risk of a toxic effect is decreased by preventing its entry into systemic circulation [8]; (c) bleeding in the surgical site is decreased because of the vasoconstrictor effect of adrenaline [9]; and (d) the vasodilator effect of the LA is decreased [10]. However, there are also some randomized controlled studies that show the ineffectiveness of LA during tonsillectomy [11]. However, a very small amount of adrenaline combined with an LA can lead to arrhythmia, uncontrolled hypertension (severe headache, blurred vision, buzzing in the ears, anxiety, confusion, chest pain, and shortness of breath), cardiogenic shock, and even myocardial infarction [12].

In our study, we primarily aimed to assess the effects of levobupivacaine and of levobupivacaine + adrenaline administered during tonsillectomy on postoperative pain and secondarily on nausea and vomiting, amount of bleeding, time to first oral intake and quality of oral intake, time to first analgesic request, and total amount of analgesics consumed until the time of discharge.

## 2. Materials and Methods

The study was undertaken with the prior approval of the Selcuk University, Department of Medical Ethics (register number: SUMF 2015-9) and the consent of the parents, as well as of patient older than six years. We used G Power software to determine the sample size. We calculated that we would need a total of 88 children (44 children for each group) to compare the postoperative pain within the two groups with 90% power, 5% type I error level, and 25% effect size for the *F* test. We enrolled 90 children to account for the possibility of exclusion. A total of 90 American Society of Anaesthesiologists class 1 or 2 (ASA I-II) patients between the ages of five and twelve participated in the study. Patients who were known to be allergic to the drugs used in the study; patients with bleeding disorders or liver, kidney, cardiac, or lung diseases; patients more than 35 kg (potentially obese or having sleep apnea); patients who used antiemetics, analgesics, steroids, or antihistamines 24 hours before the operation; patients for whom the drugs used in the study were contraindicated; patients who returned for treatment of bleeding or any other complication after the operation; or patients whose parents did not consent to their participation in the study were excluded.

We recorded patient demographic data, such as sex, age, and weight. For each patient taken into the operating room, 25–30 mL·kg^−1^ of Ringer's lactate infusion was started after venous vascular access was obtained via 22–24-gauge intravenous cannula. Premedication was performed in all patients 30 minutes before the operation with 0.5 mg·kg^−1^ of midazolam. In line with the study protocol, anaesthetic induction was started via sevoflurane mask in patients between the ages of five and seven and with propofol in patients between the ages of seven and twelve. Afterward, the patients were administered fentanyl 0.5 mg·kg^−1^ and rocuronium bromide 0.6 mg·kg^−1^. After intubation, each patient was administered 0.5 mg·kg^−1^ of dexamethasone intravenously via a suitable endotracheal intubation tube.

After anaesthetic induction and before the operation, the patients were divided into groups by using the randomization program in SPSS 20.0 statistical software. Randomization was performed by using the “Random Sample” menu in SPSS 20.0 by taking 33% of the total number of patients; we had 90 patients for the two groups.

Levobupivacaine only (0.5%) 0.4 mg·kg^−1^ for group L (*n* = 45) and levobupivacaine (0.5%) 0.4 mg·kg^−1^ + adrenaline (1 : 200.000) for group LA (*n* = 45) were prepared for each tonsillar fossa. For each tonsillar fossa, 2.5 ml of the solution was injected as a withering and swelling submucosal infiltration in sterile conditions to the inferior and superior poles and to all areas surrounding the plicae of both tonsillar fossae by the otolaryngologist, who did not know which group was receiving which treatment. Anaesthesia was maintained by using 50% O_2_ + 50% N_2_O and 2% sevoflurane. Mean arterial pressure (MAP), heart rate (HR), peripheral oxygen saturation (SpO_2_), and end-tidal pressure of carbon dioxide (ETCO_2_) were recorded before the operation; just after the anaesthetic induction; and at the 3rd, 5th, 10th, 15th, 20th, and 25th minutes of the operation until the anaesthesia ended.

At the end of the surgical procedure, bleeding control was maintained through bipolar electrocauterization. Anaesthetics were stopped after bleeding was controlled. All of the patients were administered 10–20 *μ*g·kg^−1^ atropine and 30–50 *μ*g·kg^−1^ neostigmine, and the patients were extubated when it was believed that adequate respiration and protective reflexes were maintained. At the end of the operation, the period between anaesthetic induction and patient extubation was recorded as the duration of anaesthesia, and the period between the placement of the mouth retractor for the operation and the removal of the mouth retractor after bleeding was controlled was recorded as the duration of the surgery. The perioperative amount of bleeding was deduced by measuring the aspirator apparatus and the swabs used and calculating the weight of the physiological saline solution before and after the operation. Cautery and suture use for the patients also were recorded. The patients were monitored in the postanesthesia care unit (PACU) postoperatively. The parents were taken into the PACU to decrease postoperative anxieties that the children might feel because they were in an unfamiliar environment. If present, diplopia, hallucination, cough, facial paralysis, vocal cord paralysis, bronchospasm, and postoperative bleeding were recorded as postoperative complications. At the end of the operation, the follow-up was taken over by an anaesthesiologist who did not know the study groups. In this way, the double-blind study protocol was maintained. Postoperatively, at the 10th, 30th, and 60th minutes and the 6th, 12th, and 24th hours, the pain scores of the patients were assessed by using the Children's Hospital of Eastern Ontario Pain Scale (CHEOPS), and nausea and vomiting were assessed by measuring postoperative nausea and vomiting (PONV: 0 = no nausea or vomiting; 1 = nausea but no vomiting; 2 = vomited once in 30 minutes; 3 = two or more episodes in 30 minutes). The modified Aldrete score (MAS) was used as a recovery criterion, and a MAS greater than 8 was considered to indicate recovery. For patients with a CHEOPS score greater than 6, pain treatment was conducted with intravenous acetaminophen within the first 6 hours and oral acetaminophen after the first 6 hours at a dose of 10 mg·kg·h^−1^. The time to the first analgesic request and the total amount of analgesic administered were recorded. If the PONV score was higher than 3, patients were treated with 150 *μ*g·kg^−1^ of metoclopramide intravenously.

The statistical analyses of the study were performed using SPSS 20.0 software. Descriptive measures of continuous and categorical variables were extracted and are presented as tables and graphs. Continuous variables are presented in the form of mean ± standard deviation or median (minimum and maximum) and the frequencies and percentages of categorical variables are given. The Kolmogorov-Smirnov normality test was used for continuous variables. Group comparisons of the variables that showed a normal distribution were performed by using a one-way analysis of variance, and paired comparisons were conducted by using the Tukey honest significant difference test. A Mann–Whitney *U* variance analysis was used for discrete numerical variables that did not show a normal distribution. To see the effects of time over treatments together with trials, we used a Friedman analysis of variance test and related pairwise comparisons. The relationship between the categorical variables was determined by preparing crosstabs and using the *χ*^2^ test. In all analyses, *p* < 0.05 was accepted as statistically significant.

## 3. Results

A total of 90 patients who underwent tonsillectomy were included in our study. There was no statistically significant difference between the two groups in terms of the sociodemographic variables of sex, age, weight, type of anaesthetics, duration of anaesthesia, duration of surgery, and perioperative amount of bleeding. At the same time, there was no statistically significant difference between these two treatment groups in terms of perioperative bleeding, recovery period, and perioperative cautery and suture use (Table 1). There was no statistically significant difference in the MAP, HR, SpO_2_, and ETCO_2_ scores of the two groups for the period of the operation.

The patients' CHEOPS scores (Table 2) were obtained postoperatively at both the PACU and the otolaryngology service at the 10th, 30th, and 60th minutes and 6th, 12th, and 24th hours. There was no significant difference in pain scores between treatment groups L and LA. A significant decrease in CHEOPS score was observed at every time point until postoperatively at the 10th, 30th, and 60th minutes and the 6th, 12th, and 24th hours within the two groups (*p* < 0.001).

The comparison of the groups in terms of time to first oral intake revealed a no significant difference between groups L and LA (*p* = 0.900). There were also no statistically significant differences between groups L and LA in terms of time to first analgesic request and the total dose of analgesic requirements (*p* = 0.889) (Table 3).

There was no statistically significant difference between the two groups in terms of postoperative PONV values at the 10th, 30th, and 60th minutes and the 6th, 12th, and 24th hours. The PONV values of groups L and LA were zero at the 60th minute and at the 6th and 24th hours (Table 4).

No significant decrease in PONV was observed at any time point until postoperatively at the 10th, 30th, and 60th minutes and 6th, 12th, and 24th hours in two the groups (*p* < 0.001).

When the groups were compared in terms of postoperative complications, nausea and vomiting were observed in four patients in group L and in four patients in group LA. No statistically significant differences were found between groups L and LA. There was no statistically significant difference between the groups in terms of other postoperative complications.

## 4. Discussion

In our study, in both the levobupivacaine and the levobupivacaine + adrenaline groups, patients had the same postoperative pain scores and PONV rates, and equal amounts of analgesics were consumed up to the 24th hour. There also were the same time until first oral intake, recovery time and time to first analgesic request, and amount of bleeding.

After tonsillectomy, it is difficult to provide sufficient analgesia while protecting airway reflexes, without delaying recovery and without providing sedation. In tonsillectomies, the inadequacy of early postoperative pain control also delays mucosal healing [13].

Experimental studies assert that the changes in neural excitability as a result of sensitivity to a nociceptive stimulus and a decreased pain threshold are caused by peripheral tissue damage [1, 2, 6, 14]. The reason for using LAs perioperatively is not only to block peripheral nociceptive excitation but also to prevent the sensitization of the central nervous system. LA administration is performed before or after tonsillectomy, either topically or in the form of infiltration to the tensile fossa [5]. The intraoral route of the glossopharyngeal nerve within the posterior pillar may compromise function and has resulted in swallowing impairment. This is not as much of a problem with topical application as it has been with specific glossopharyngeal nerve block; however, transmucosal penetration of LA from its topical application may contribute to swallowing impairment [15].

In addition to intravenous, intramuscular, and oral or rectal postoperative analgesic use, various methods have been tried, such as the injection of anaesthetic substances or penicillin-steroid mixtures into the tonsillar fossa postoperatively if pain develops postoperatively [10, 14]. Although some studies show that LA infiltration decreases postoperative sore throat [3, 4, 10, 16], other studies show that it is not useful for decreasing postoperative pain [2, 4, 14, 17]. The reasons for these differences are said to be the steroid and additional sedative drugs used in premedication, the technique of tonsillectomy, the technique of peritonsillar injection, volume and dose of LA, and postoperative pain evaluation methods.

In children, measuring pain severity is important for its treatment and follow-up. Because the cognitive and verbal communication skills of children are not adequate, it is difficult to provide a correct assessment of pain. For this reason, follow-up of findings by means of standard parameters should produce success in diagnosing and effectively treating pain [18]. In our study, we endeavoured to provide an accurate assessment by using the CHEOPS.

Karaaslan et al. showed that the use of levobupivacaine on its own and levobupivacaine + magnesium produced low CHEOPS scores [19]. In contrast, Tas et al. found that postoperative pain control was better in patients in whom they had administered levobupivacaine + adrenaline than in a control group [20]. In the present study, we found that use of levobupivacaine + adrenaline is not superior to use of levobupivacaine alone in reducing postoperative pain.

Although some surgeons prefer perioperative infiltration to observe possible perioperative bleeding more easily, others prefer to see whether there is perioperative bleeding of the tonsils intraoperatively without using an intraoperative injection. For this reason, some otolaryngologists do not use perioperative infiltrative agents. However, others prefer the perioperative infiltrative use of vasoconstrictive drugs, such as adrenaline, and have reported that they have encountered less perioperative bleeding [12, 21]. In contrast, there are studies stating that there are no significant differences in the amount of perioperative bleeding between control groups and groups that have received an LA + adrenaline [22] and that vasoconstrictor effects of levobupivacaine at low concentrations are as effective as those of levobupivacaine + adrenaline [23]. In this study, we found no significant differences between the two groups in the amount of bleeding.

Insufficient pain treatment may increase morbidity after certain operations such as tonsillectomy. PONV incidence, which occurred in 40% of patients with postoperative pain, decreased to 16% in patients who underwent treatment for pain. The role of corticosteroids in PONV prophylaxis is known [24]. It is necessary to avoid risk factors as much as possible to decrease nausea and vomiting rates to a minimum. Risk factors are the use of nitrous oxide, volatile agents, high-dose uploads, inadequate hydration, anxiety, and insufficient pain treatment [25, 26]. For this reason, in this study we aimed to achieve standardization in the midazolam dose in the preoperative premedication and in fentanyl and dexamethasone doses in the perioperative anaesthesia. In their study, Cocelli et al. showed that the rate of nausea and vomiting was lower in the LA group than in the control group [2]. In our study, the PONV rates of both groups were equal in the recovery room (first 30 minutes postoperatively), and there was no difference between groups L and LA 24 hours after recovery. We believe that less use of cautery and suture decreased the sensitivity to nausea and vomiting.

Bipolar cauterization of bleeding points to treat perioperative bleeding causes excessive tissue damage in the tonsillar bed and increases postoperative sore throat. Postoperative pain not only causes fear and discomfort but also affects the time to first oral intake. In our study, oral intake started at the end of the first postoperative hour in groups L and LA and continued to progress until 24 hours postoperatively in both groups. We believe that this was due to less use of cautery and suture in groups L and LA.

Pain is often severe after tonsillectomy and may require the use of opioid analgesics. Although opioids are seen as a remedy for pain, they have serious side effects such as respiratory depression, sedation, and nausea and vomiting. Another frequently used medication for stopping pain after tonsillectomy is nonsteroid anti-inflammatory drug. However, the use of these agents may increase postoperative bleeding. For this reason, in our study, we used acetaminophen for postoperative pain relief. In a study that presented the dependence of time to first analgesic request on perioperative LA infiltration, Basuni et al. measured the time to first analgesic request to be 373.2 ± 63.6 minutes when they used levobupivacaine [27]. Kasapoglu et al. found a significant difference between their levobupivacaine group and their control group in terms of the time to first analgesic request [3]. In our study, similar to the findings of Basuni et al., the times to first analgesic request in groups L and LA were 379.1 ± 172.3 and 383.8 ± 173.5 minutes, respectively [27]. In our opinion, these times were longer because levobupivacaine is a long-acting LA, but levobupivacaine with the addition of adrenaline did not alter the analgesic requirement time.

The total dose of additional analgesics in the postoperative period was the same for groups L and LA. The combination of levobupivacaine + adrenaline did not change the effective time of the LA.

Peritonsillar infiltration, although a simple technique in skilled hands, has been associated with major morbidity. The possible complications have been described in a report of more than 1,000 patients receiving a mixture of lidocaine, methyl prednisolone, and penicillin. Possible complications related to it included advertent intravascular or intra-arterial (carotid artery) injection leading to central nervous system (grand mal seizure) or cardiovascular toxicity, hemorrhage, airway obstruction, allergy, vocal cord paralysis, and mucosal sloughing [28]. Infiltration presents the risk of delivery to the vein by mistake, which may cause convulsion and cardiac arrest. Intravascular (especially intra-arterial) injection of LA can be lethal [29]. It also carries the risks of upper airway obstruction, facial paralysis, and vocal cord paralysis. However, no complications were observed related to preoperative levobupivacaine + adrenaline or levobupivacaine infiltration in our study. There were no significant differences between the groups in terms of cough and bronchospasm, which are regarded as postoperative complications. Postoperative cough and bronchospasm were treated with cold vapor, and vomiting was treated with 150 *μ*g·kg^−1^ intravenous metoclopramide in the PACU and the otolaryngology ward.

There were some limitations to our study. First, the otolaryngologists wanted to hospitalize the postoperative tonsillectomy patients for at least one night and monitor postoperative complications; discharge periods of the two groups could not be compared. Second, the consecutive pain measurement (CHEOPS) has taken a minimum score (4) as baseline pain measurement not to cause preoperative anxiety in the children.

## 5. Conclusion

Levobupivacaine or levobupivacaine + adrenaline infiltration into the tonsillar fossa before pediatric tonsillectomy did not cause any changes in postoperative pain, time to first oral intake, total amount of analgesics used and recovery period, time to first analgesic request, and amount of bleeding. For this reason, perioperative levobupivacaine infiltration alone can be used without causing any of the well-known side effects of adrenaline in pediatric tonsillectomy.

## Figures and Tables

**Table 1:** The demographic and operative data and comparative variables between the two groups and values given are mean ± st.

	Group L Levobupivacaine (*n* = 45)	Group LA Levobupivacaine + adrenaline (*n* = 45)	*p*
Gender, male/female	15/14	16/13	0.546
Age (year)	7.1 ± 2.3	7.0 ± 2.1	0.574
Weight (kg)	23 ± 7	22.3 ± 5.5	0.882
Anesthesia type1^*∗*^/2^†^	15/14	15/14	0.532
Duration of anaesthesia (min)	28.2 ± 5.4	27.8 ± 4.2	0.329
Duration of surgery (min)	15.5 ± 4.2	16.9 ± 5.2	0.508
Recovery time (min)	19.7 ± 7.0	18.3 ± 6.9	0.639
Discharge time (h)	26.4 ± 1.1	26.8 ± 1.3	0.996
Volume of perioperative bleeding (ml)	20.7 ± 6.4	19.8 ± 5.3	0.575
Cautery+/−	11/18	9/20	0.595
Suture+/−	2/27	1/28	0.557

min: minute, h: hour, +: patients who used cautery/suture, and −: no patients who used cautery/suture. ^*∗*^Patients who used volatile anesthetic for induction of anesthesia. ^†^Patients who used propofol for induction of anesthesia.

**Table 2:** The CHEOPS variables of three groups at each time interval after operation and values given are median (min–max).

	Group L Levobupivacaine (*n* = 45) Med (min–max)	Group LA Levobupivacaine + adrenaline (*n* = 45) Med (min–max)	*p*
CHEOPS 10th min	5 (4-8)	5 (4-8)	0.801
CHEOPS 30th min	4 (4-8)^*∗*^	4 (4-5)^*∗*^	1
CHEOPS 60th min	4 (4-4)^*∗*†^	4 (4-4)^*∗*†^	1
CHEOPS 6th h	4 (4-5)^*∗*†‡^	4 (4-6)^*∗*†‡^	0.966
CHEOPS 12th h	4 (4-5)^*∗*†‡§^	4 (4-5)^*∗*†‡§^	0.966
CHEOPS 24th h	4 (4-5)^*∗*†‡§*∗∗*^	4 (4-5)^*∗*†‡§*∗∗*^	1

CHEOPS: Children's Hospital of Eastern Ontario Pain Scale, min: minute, and h: hour. ^*∗*^When compared with CHEOPS 10th within group (*p* < 0.001). ^†^When compared with CHEOPS 30th within group (*p* < 0.001). ^‡^When compared with CHEOPS 60th within group (*p* < 0.001). ^§^When compared with CHEOPS 6th within group (*p* < 0.001). ^*∗∗*^When compared with CHEOPS 12th within group (*p* < 0.001).

**Table 3:** The postoperative data and comparative analysis between the two groups.

	Group L Levobupivacaine (*n* = 45) Med (min–max)	Group LA Levobupivacaine + adrenaline (*n* = 45) Med (min–max)	*p*
Time to first oral intake (min)	119 (50-265)	115 (50-253)	0.900
Time to first analgesic request (min)	350 (120-781)	350 (118-801)	0.889
The total dose of analgesic requirements (mg)	28 (23.4-33.5)	30 (24.7-35.2)	0.501

min: minute and mg: milligram.

**Table 4:** The PONV variables of two groups at each time interval after operation and values given are median (min–max).

	Group L Levobupivacaine (*n* = 45) Med (min–max)	Group LA Levobupivacaine + adrenaline (*n* = 45) Med (min–max)	*p*
PONV 10th min	0 (0-1)	5 (4-8)	0.801
PONV 30th min	4 (4-5)	4 (4-5)	1
PONV 60th min	4 (4-5)	4 (4-4)	1
PONV 6th h	3 (1-4)	4 (4-5)	0.966
PONV 12th h	3 (2-4)	4 (4-6)	0.966
PONV 24th h	4 (4-5)	4 (4-5)	1

PONV: postoperative nausea and vomiting, min: minute, and h: hour.
